# Electrophilic Reactivity of Sulfated Alcohols in the Context of Skin Sensitization

**DOI:** 10.1021/acs.chemrestox.3c00292

**Published:** 2023-12-11

**Authors:** David W. Roberts

**Affiliations:** School of Pharmacy and Biomolecular Sciences, Liverpool John Moores University, Liverpool L3 3AF, U.K.

## Abstract

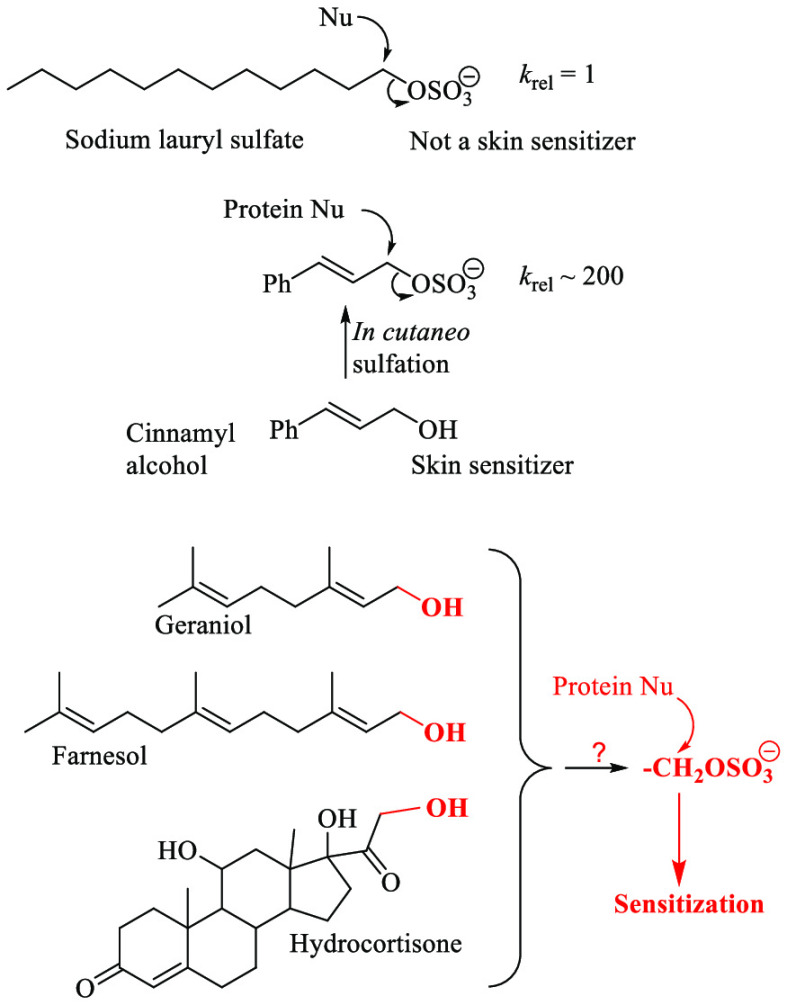

The surfactant sodium lauryl sulfate (SLS), although consistently positive in the murine local lymph node assay (LLNA) for skin sensitization, shows no evidence of being a human sensitizer and is often described as a false positive, lacking structural alerts for sensitization. However, there is evidence of the cinnamyl sulfate anion being the metabolite responsible for the sensitization potential of cinnamyl alcohol to humans and in animal tests. Here, manufacturing chemistry data and physical organic chemistry principles are applied to confirm that SLS is not reactive enough to sensitize, whereas sensitization to cinnamyl alcohol via cinnamyl sulfate is plausible. Sensitization data for several other primary alcohols, including geraniol, farnesol, and possibly hydrocortisone, are also consistent with this mechanism. It seems possible that biosulfation may play a wider role than has previously been recognized in skin sensitization.

Sodium lauryl sulfate (SLS) is a widely used surfactant with a long history of use in consumer products without any indications of the ability to cause skin sensitization in humans. However, it is known to consistently give positive results in the murine local lymph node assay (LLNA).^1,2^ The LLNA is designed to quantify sensitization potency in terms of the EC3 value, this being the concentration of test chemical that when applied to the mouse ear gives a 3-fold increase in lymphocyte proliferation in the draining lymph node.^3^ Skin sensitizers are in most cases either directly reactive as electrophiles or able to act as precursors of electrophilic species, and act via covalent modification of protein nucleophiles such as cysteine units.^4,5^ SLS is usually regarded as a false positive and it is often stated in the skin sensitization literature (e.g., ref (2)) that SLS has no alerts for reactivity. However, the −OSO_3_^–^ group is not completely unreactive, and indeed it has been argued, based on experimental evidence (*vide infra*), that skin sensitization to cinnamyl alcohol involves activation in the skin by metabolic conversion to cinnamyl sulfate which reacts with protein nucleophiles.^6^ It is, therefore, appropriate to consider the reactivity of sulfated alcohols in the context of skin sensitization.

It is not completely accurate to describe SLS as having no reactivity alerts. The −OSO_3_^–^ group is a recognized S_N_2 leaving group,^7^ and, in addition to its use *per se* as a surfactant, SLS can be used as an electrophilic intermediate in the chemical industry, for example in the manufacture of amine oxide surfactants.^8^ However, in these applications, the reaction temperatures required are substantially higher than physiological temperature.

A 1983 European patent application^8^ gives an example with quantitative detail enabling rough estimates of kinetic data to be made. The patent application describes the use of SLS in the production of amines and gives a detailed example of the reaction with dimethylamine, catalyzed by sodium iodide. The reaction can be summarized:
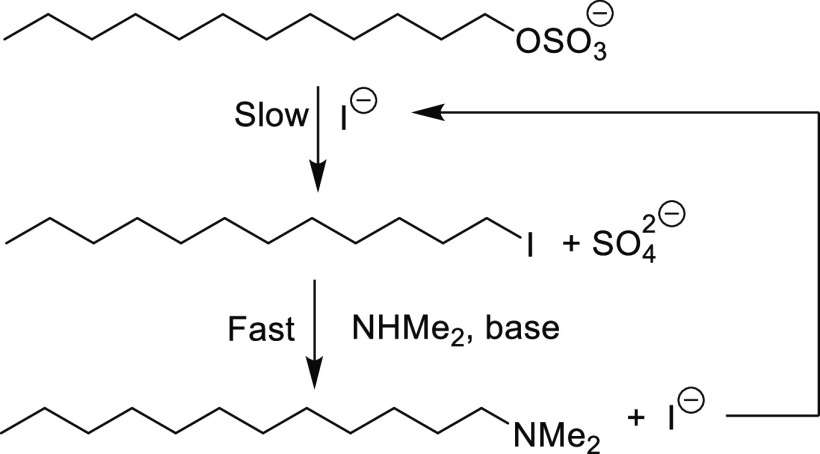


The rate-determining step is the S_N_2 reaction between SLS and iodide ion, whose nucleophilic constant value (5.04)^9^ is similar to that of cysteine (5.1).^10^ The reaction corresponds to pseudo first order in SLS, the iodide ion concentration being constant. The example given in the patent states: To a 1 L stirred autoclave, 125 g of sodium dodecyl sulfate (0.434 mol), 17.4 g of sodium hydroxide (0.434 mol), 250 g of water (as solvent, 13.88 mol), 280 g of dimethylamine (6.22 mol), and 32 g of sodium iodide (0.21 mol) are added and heated to a temperature of 154.4 °C and a pressure of 500 psi (35.1 kg./sq.cm.). After 30 min of reaction time, conversion is 67.3% to a product that is 98.2% dimethyldodecylamine.

From these figures, the iodide ion concentration in the reaction mixture can be estimated as ∼0.4M. From the figure of 67.3% conversion after 30 min the pseudo first order rate constant can be estimated and divided by the iodide concentration to give an estimated second order rate constant of 1.55 × 10^–3^ s^–1^ M^–1^ for the reaction of SLS with iodide ion at 154.4 °C. This estimate is of course very approximate, since the reaction conditions are far from ideal for kinetic studies. Two major complicating features are: very high concentrations of the reagents; possibility of micelle formation (increasingly likely as the reaction proceeds since incorporation of the reaction product into the micelles will reduce the critical micelle concentration), which would make the concentration of SLS less than nominal. Also, heating of the reaction mixture from ambient to nominal reaction temperature will account for a significant proportion of the nominal reaction time. Consequently, the above value for the rate constant is more likely to be an underestimate than an overestimate.

The activation energy for the reaction does not appear to have been reported in the literature, but by comparison with other S_N_2 reactions, it can be assumed to be in the range 12–16 kcal/mol. For example, the activation energy for the S_N_2 reaction of styrene epoxide with piperidine is 14 kcal/mol.^11^ Within this activation energy range, rate constants for reaction of SLS with iodide ion at 25° can now be estimated and, based on similar nucleophilicity values of the ionised cysteine unit and iodide ion,^7,8^ the LLNA potency (EC3) for SLS can be predicted from a quantitative mechanistic model (QMM) relating pEC3 of S_N_2 electrophiles to a combination of log*k* and log*P*,^12^ using the calculated log*P* value of 1.6 for SLS.^13^ Results of these calculations are shown in Table 1. The equations used for these calculations are:

**Table 1:** Estimated Rate Constants at 25° and Predicted EC3 Values for SLS

Assumed *E*_act_ (kcal/mol)	Log*k* (*k* in s^–1^ M^–1^)	Predicted EC3 (%)
16	–6.40	5200
15	–6.15	3600
14	–5.92	2600
13	–5.70	1800
12	–5.47	1300
Range of nine experimental EC3 values^14^		1.5–17.1

Arrhenius equation:

For present purposes the Arrhenius equation,^11^ ln*k* = ln*A* – *E*_act_/*RT*, can be expressed as
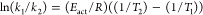
1where *k*_1_ and *k*_2_ are the rate constants at temperatures *T*_1_ and *T*_2_, respectively, *E*_act_ is the activation energy in calories per mole, and *R* is the gas constant (= 1.987 when *E*_act_ is expressed in calories). To apply eq 1, temperatures must be converted to degrees K, by addition of 273 to the temperature in degrees centigrade, i.e., 25 °C = 298 K.

QMM for S_N_2 electrophiles:^12^
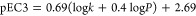
2

The predicted EC3 values are all very much higher than 100%, predicting SLS to be a nonsensitizer. For a positive sensitization prediction, a highly unrealistic activation energy <5 kcal/mol would have to be assumed. It can be concluded with confidence that although SLS is electrophilic, it is not reactive enough to sensitize.

These calculations are consistent with the view that SLS is a nonsensitizer, and its LLNA result is a genuine false positive. SLS has been discussed in depth by Basketter et al.^2^ It is an irritant, but irritancy in general is not significantly correlated with sensitization potency and there is still no clear mechanistic explanation as to why SLS is positive in the LLNA.

Cinnamyl alcohol is a known skin sensitizer, with an EC3 of 21%.^14^ Evidence from HR-MAS NMR studies with reconstituted human epidermis (RHE)^6^ suggests that it sensitizes via *in cutaneo* conversion to the electrophilic cinnamyl sulfate PhCH=CHCH_2_OSO_3_^−^. The seemingly simpler explanation, that it sensitizes via oxidation to the known sensitizer cinnamic aldehyde, seems less likely in light of clinical observations that many patients sensitive to cinnamyl alcohol do not react to cinnamic aldehyde.^15^ Although cinnamyl alcohol subjected to air exposure has been shown to become more antigenic (EC3 4.9%) due to formation of its epoxide together with cinnamic aldehyde, both of which are strong sensitizers in the LLNA,^16^ protein-binding products derived from these compounds were not observed in the RHE-NMR studies with pure cinnamyl alcohol.^6^ Based on the calculations for SLS reactivity, the question of whether cinnamyl sulfate is reactive enough to rationalize the sensitization potency of cinnamyl alcohol can now be addressed.

The cinnamyl group may be regarded as a vinylogous benzyl group. S_N_2 reactions at a benzyl group are about 200-times faster than reactions at a primary alkyl group.^7^ This is consistent with findings of Nayami et al.^17^ that PhCH=CHCH_2_Cl is 10^2^–10^3^ times as reactive as PhCH_2_CH_2_Cl.

Applying the factor of 200 to the rate constants estimated for SLS, rough estimates of the rate constant can be made for cinnamyl sulfate. Applying the S_N_2 QMM^12^ (eq 2), using these estimates of the rate constant for cinnamyl sulfate and the log*P* value of cinnamyl alcohol (published values are in the 1.45 to 1.95 range^18,19^), the predicted EC3 values shown in Table 2 are obtained.

**Table 2:** Estimated Rate Constants for Cinnamyl Sulfate and Predicted LLNA Potency for Cinnamyl Alcohol Based on Its *In Cutaneo* Activation to Cinnamyl Sulfate

Assumed *E*_act_ (kcal/mol) for SLS	Estimated log*k* (*k* in s^–1^ M^–1^) for cinnamyl sulfate	Predicted EC3 (%) based on log*P* range 1.45–1.95
16	–4.1	50–70
15	–3.8	35–48
14	–3.6	25–34
13	–3.4	17–23
12	–3.2	12–16
Experimental LLNA EC3 for cinnamyl alcohol^6^		21

The predicted EC3 values within the likely range of activation energies for SLS are consistent, based on activation by *in cutaneo* sulfation, with the observed weak sensitization potency of cinnamyl alcohol.

Some other primary alcohols that might act as skin sensitizers via metabolic activation to their sulfates are considered in Table 3. In each case, two estimated log*k* values for the sulfated derivatives are considered, derived from the highest and lowest log*k* estimates in Table 1 for SLS by addition of log(167) or log(66) for benzylic activation or allylic activation, respectively, 167 and 66 being taken as the rate constants for reaction at benzyl carbon or allylic carbon respectively relative to reaction at 1-dodecyl carbon.^7^ Predicted EC3 values are calculated from the log*P* values of the alcohols (calculated manually by the method of Hansch and Leo^20^) and the estimated log*k* values for their sulfates, using eq 2. It may be noted that sodium lauryl sulfate being a nonsensitizer does not of itself preclude lauryl alcohol from being a sensitizer via metabolic conversion to its sulfate.

**Table 3:** Predicted EC3 Values for Primary Alcohols Based on Metabolic Activation to Their Sulfates

Alcohol	Log*P*	Log*k* range	Pred. EC3 (%)	Observed potency
Lauryl alcohol	5.19	–5.47 to −6.4	83–366	No LLNA data. Not classified as a sensitizer
C_12_H_25_OH				
Benzyl alcohol	1.10	–3.25 to −4.18	19–84	EC3 > 50%.^21^ Human potency category^c^ 4^22^
PhCH_2_OH				
Geraniol^a^	2.75	–3.65 to −4.58	18–79	EC3, 26%.^1^ Human potency category 4^22^
Farnesol^b^	4.77	–3.65 to −4.58	7.2–32	EC3, 5.5%.^23^ Human potency category 3^22^

a Geraniol: Me_2_C=CH(CH_2_)_2_C(Me)=CHCH_2_OH

b Farnesol: Me_2_C=CH(CH_2_)_2_C(Me)=CH(CH_2_)_2_C(Me)=CHCH_2_OH

c Chemicals with human sensitization information have been classified into six human potency categories^24^ ranging from 1 (extreme sensitizers) to 6 (nonsensitizers). Category 5 sensitizers are treated for regulatory purposes as nonsensitizers; category 4 sensitizers can be considered as weak sensitizers and are defined as requiring considerable/prolonged exposure to higher dose levels to produce sensitization, which even then is unlikely to exceed 0.01% of those exposed; category 3 sensitizers can be considered as moderate sensitizers and are defined as substances that may be quite well-known as contact allergens, but for which a substantial degree of exposure typically is necessary to produce sensitization in 0.01% to 0.1% of those exposed.

The lack of significant potency of lauryl alcohol, the weak sensitization potency of benzyl alcohol and geraniol, and the moderate sensitization potency of farnesol are all consistent with metabolic activation by sulfation. The sulfates of geraniol and farnesol are not expected to be significantly different in reactivity, and the higher potency of farnesol relative to those of geraniol and cinnamic alcohol is attributable to farnesol having a higher log*P* value.

Carbonyl groups can have substantially larger activating effects than benzyl or allylic groups. The rate constant for chloroacetone is 30,000-times larger than that of 1-dodecyl chloride reacting with iodide ion.^7^ Thus, a ketosulfate RCOCH_2_OSO_3_^–^ would be about 30,000-times as reactive as SLS. The −COCH_2_OH substructure is consequently a potential alert for sensitization via metabolic sulfation of the OH group. However, compounds with this substructure might also sensitize directly by nucleophilic addition to the carbonyl group (if the −CO– unit is a ketone group, in which case its electrophilicity is enhanced by the electronegativity of the CH_2_OH group) or via oxidation of the −CH_2_OH unit to −CHO followed by nucleophilic addition to the aldehydic carbonyl group. The latter mechanism has been proposed for hydrocortisone and other corticosteroids for which clinical evidence of skin sensitization has been reported.^25^

These corticosteroids are all α-hydroxyketones having a −COCH_2_OH group bonded to a ring carbon atom at position 17 of a steroid ring structure. Since these corticosteroids have immunosuppressive properties, animals cannot be readily sensitized,^25^ and there is a lack of meaningful data that would enable potency to be compared against that of other types of sensitizers. The chemical mechanism of sensitization by corticosteroids remains an open question. From the log*P* value of 1.61^26^ and log*k* values ranging from −1.92 to −0.99 (derived by adding log(30,000) to the log*k* range of −6.4 to −5.47 for SLS), it can be calculated from eq 2 that hydrocortisone would have an EC3 value in the range 1.3 to 5.7% if activated by sulphation and if it were not immunosuppressive.

For comparison, EC3 values calculated for hydrocortisone acting as a Schiff base electrophile either directly or by reaction of the −CHO group resulting from oxidation of the −CH_2_OH group are 36% or 0.7% respectively. These values are calculated from the Schiff base QMM^27^ (pEC3 = 1.12Σσ* + 0.42 log*P* – 1.62, where Σσ* is the sum of the Taft substituent constants for the groups bonded to the reacting carbonyl group) using the log*P* value of 1.61 for hydrocortisone^26^ and Σσ* values, estimated by the methods described by Perrin, Boyd, and Serjeant^28^ of 0.85 or 2.39 for direct reaction or reaction after oxidation, respectively.

These estimated EC3 values are of course hypothetical since in practice, due to the immunosuppressive properties, the LLNA would be inapplicable for hydrocortisone.

In conclusion, manufacturing chemistry data for production of amines from sulfated alcohols have been applied: (a) to confirm that the observed positive result for SLS in the LLNA is a false positive and cannot be attributed to the electrophilic properties of SLS and (b) to support the mechanism proposed by Moss et al.,^6^ based on *in cutaneo* sulfation, for the weak LLNA potency of cinnamyl alcohol. Similar calculations support, though they do not prove, a similar mechanism for skin sensitization by other primary alcohols with neighboring unsaturated groups, such as geraniol, farnesol, and hydrocortisone.

Bearing in mind that sulfotransferases are present in the skin at relatively high concentrations^29^ and that there is evidence for their involvement in drug-induced skin rashes,^30^ it seems possible that biosulfation may play a wider role than has previously been recognized in skin sensitization.
